# Population growth as a driver of initial domestication in Eastern North America

**DOI:** 10.1098/rsos.160319

**Published:** 2016-08-03

**Authors:** Elic M. Weitzel, Brian F. Codding

**Affiliations:** Department of Anthropology and Archaeological Center, University of Utah, 270 S. 1400 E., Rm. 102, Salt Lake City, UT 84112, USA

**Keywords:** origins of agriculture, niche construction, behavioural ecology, optimal foraging theory, dates as data

## Abstract

The transition to agriculture is one of the most significant events in human prehistory; yet, explaining why people initially domesticated plants and animals remains a contentious research problem in archaeology. Two competing hypotheses dominate current debates. The first draws on niche construction theory to emphasize how intentional management of wild resources should lead to domestication regardless of Malthusian population–resource imbalances. The second relies on models from behavioural ecology (BE) to highlight how individuals should only exert selective pressure on wild resources during times of population–resource imbalance. We examine these hypotheses to explain the domestication event which occurred in Eastern North America approximately 5000 years ago. Using radiocarbon date density and site counts as proxies for human population, we find that populations increased significantly in the 1000 years prior to initial domestication. We therefore suggest that high populations prior to 5000 cal BP may have experienced competition for and possibly overexploitation of resources, altering the selective pressures on wild plants thereby producing domesticates. These findings support the BE hypothesis of domestication occurring in the context of population–resource imbalances. Such deficits, driven either by increased populations or decreased resource abundance, are predicted to characterize domestication events elsewhere.

## 1. Introduction

Explaining domestication remains one of the most important and contentious research problems in archaeology [1–3]. Current explanations often take one of two approaches. The first hypothesis draws on the concept of niche construction (NC) to emphasize that intentional experimentation with and management of wild resources should lead to domestication regardless of Malthusian [4] population–resource imbalances [2,3,5]. The other hypothesis is derived from behavioural ecology (BE) and, based on predictions from foraging theory models, highlights how individuals should only exert selective pressure on wild resources during times of Malthusian population–resource imbalance, which encourage individuals to intensify their economies [6,7] in order to efficiently acquire more food from the environment [1,8–10]. Thus, the NC framework predicts that domestication should occur during times of stable populations and high resource abundance, while the BE framework predicts that domestication should only occur following periods of population growth or declines in the availability of profitable resources. For simplicity, we refer to these respectively as the NC and BE hypotheses throughout this paper.

These competing explanations for the origins of domestication are at the centre of an ongoing debate in Eastern North America where, beginning about 5000 years ago, individuals began to domesticate the series of plant species that would come to constitute the Eastern Agricultural Complex. Squash (*Cucurbita pepo*) was the first of these species to be domesticated, with a date of 5025 cal BP from the Phillips Spring site in Missouri [11,12]. This date represents the earliest evidence of domestication in North America. At the Hayes site in Tennessee, domesticated sunflower seeds (*Helianthus annus* var. *macrocarpus*) were dated to 4840 cal BP [11–13]. Marshelder (*Iva annua* var. *macrocarpa*) was seemingly domesticated next, with a date of 4400 cal BP from the Napoleon Hollow site in Illinois [11,12]. Evidence for domestication from these three sites consists of small, isolated finds of botanical remains, making discussion of a crop complex difficult until 3800 cal BP, when chenopod (*Chenopodium berlandieri*), the last of the four morphologically changed domesticates added to the Eastern Agricultural Complex, was identified in large quantity from the Riverton site in Illinois, along with remains of squash, sunflower and marshelder [12]. This date from Riverton just barely edges out what were previously the earliest dates for domesticated chenopod from the Cloudsplitter and Newt Kash rockshelters in Kentucky, 3700 and 3640 cal BP, respectively [11]. Another site with early evidence of domesticates is the Marble Bluff site in Arkansas, with substantial amounts of stored squash, marshelder, sunflower and chenopod dating to 3400 cal BP. These latter four sites are argued to represent the beginnings of a domesticated crop complex due to the representation of all four domesticated plants in considerable quantities [12].

**Figure 1.**
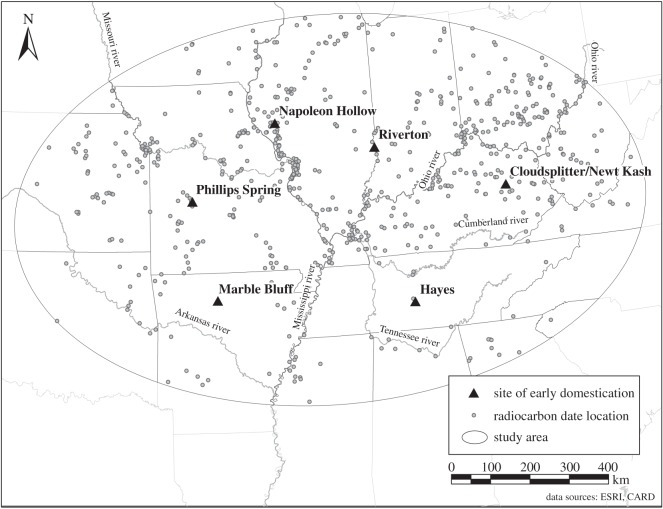
Map of the study area bounded by a standard deviational ellipse (*α* = 0.05) based on the seven sites with the earliest dated evidence of domesticates in Eastern North America and the locations of radiocarbon dates sampled from the CARD [18].

In line with BE logic, we predict that disequilibrium between populations and resources provided the incentives for individuals to either intentionally or unintentionally domesticate wild plants in Eastern North America. To test this prediction, we focus our analysis on population change as a driver of this disequilibrium. The NC hypothesis of initial domestication explicitly predicts that initial domestication in Eastern North America occurred in a context of low population density, while ‘evidence of population growth just prior to or concomitant with the initial appearance of domesticates … would support the [BE] hypothesis’ [2, p. 241]. While relative measures of human populations are difficult to acquire archaeologically, recent research has made progress using summed probability distributions of radiocarbon dates as proxies for human population levels [14–17]. Following this approach, we use radiocarbon date densities and site counts to determine whether or not significant population growth occurred prior to or during initial domestication in the region surrounding those sites which provide the earliest evidence of domestication in Eastern North America (figure 1). If the NC hypothesis of domestication is correct, then there should be evidence of low and unchanging population densities prior to initial domestication at 5000 cal BP, indicating that individuals domesticated plants through experimentation in times of relative plenty. If the BE hypothesis is correct, then there should be evidence of relatively high and increasing population densities before initial domestication, indicating that individuals domesticated plants out of necessity during times of relative scarcity.

## 2. Material and methods

To generate and evaluate the population proxies used in this paper, we undertook a seven-step process wherein we: (1) defined the study area, (2) queried the radiocarbon record and removed spurious dates, (3) calibrated the dates, (4) described the temporal distribution of dates following two approaches, (5) corrected each distribution for taphonomic bias, (6) fitted the resulting data with a statistical model and (7) evaluated the rate of change in the model fit to identify significant periods of population growth or decline. Each of these steps is detailed below.

### 2.1. Defining the study area

The study area for this research was defined according to the seven sites identified by Smith [19] which provide the earliest dated evidence of domesticates in Eastern North America: Cloudsplitter, KY; Newt Kash, KY; Hayes, TN; Marble Bluff, AR; Philips Spring, MO; Napoleon Hollow, IL; and Riverton, IL. A standard deviational ellipse [20,21] was constructed at 2 s.d. (*α* = 0.05) to define the study area according to the central tendency, dispersion and directional trend of the distribution of these seven early sites (figure 1) [21]. The standard deviational ellipse method calculates the mean centre point of the input data based on the spatial coordinates of these locations. It also determines *x*- and *y*-axes based on the directional trends of the input data, which are then used, along with the mean centre, to calculate standard deviational distances along these axes for which 95% (at *α* = 0.05) of the statistical population from which the sampled input coordinates are drawn should be enclosed within the generated ellipse.

### 2.2. Querying and cleaning the radiocarbon data

With our study area defined in this manner, we queried the Canadian Archaeological Radiocarbon Database (CARD) [18] on 25 November 2015 to obtain radiocarbon dates within the bounds of our study area. While the CARD contains an incomplete and regionally biased sample of radiocarbon dates due to differential reporting of dates and non-random excavation and dating of sites, radiocarbon dates throughout most of the study area are numerous and spread across much of the region. To ensure chronometric hygiene, we made certain that (1) any duplicate dates (based on laboratory numbers) were removed, (2) all dates originating from palaeobiological and geological contexts were removed from our dataset and (3) all dates marked as ‘anomalous' in the CARD were eliminated. Despite well-known problems with ‘old dates’, dates on different organic materials, and dates with large standard errors, further radiocarbon dates were not removed based on their age or standard error to ensure transparency, simplicity and replicability of our results and to avoid making any assertions concerning initial human colonization of the region. While we cannot provide the data from the CARD as it contains sensitive site location information, it is publicly accessible to individuals whose credentials have been vetted by the CARD administrators [18].

### 2.3. Calibration

The radiocarbon dates resulting from our standard deviational ellipse query and chronometric hygiene decisions were then calibrated using OxCal v. 4.2 according to the IntCal13 calibration curve [22]. Using the median values of these calibrated dates, we adopted the ‘dates as data’ approach [23] formalized using two methods.

### 2.4. Summarizing chronological distributions

The first approach describes the distribution of dates through kernel density estimation (KDE). The second defines the number of occupied (dated) sites per a specified interval of time using a binning or histogram approach. To implement the first approach, we generated kernel density estimates using all median calibrated dates following the Sheather–Jones method [24]. The KDE approach is similar to the summed radiocarbon probability distribution approach [17], but does not incorporate the complete calibrated distribution. The resulting values were then extracted for every 100 year interval between 15 000 cal BP and the present. To implement the second approach, we determined the number of sites with dates falling within 100 year intervals between 0 and 15 000 cal BP. Possible redundancies and inaccuracies in the dataset were eliminated by using only sites with complete Smithsonian Trinomials listed in the CARD. This provides a semi-independent line of evidence for population changes proposed by Williams [17].

### 2.5. Taphonomic correction

To account for the greater probability that older material will be lost, we applied the taphonomic correction developed by Surovell *et al.* [25] (electronic supplementary material, figure S1). While this correction is not universally accepted, it is the best available method to account for the increased likelihood of loss of sites and materials with increased time since deposition. We also made use of uncorrected densities and counts of radiocarbon dates to ensure that our results were not produced, amplified or dampened solely by the taphonomic correction process. To assess the effects of sample size and overlap between observed and corrected dates, we calculated 95% confidence intervals via a bootstrapping method, sampling 50% of the database over 1000 iterations (electronic supplementary material, figure S1). This process resulted in four population proxies: observed and corrected radiocarbon frequencies described via KDE and observed and corrected number of dated sites described via histogram. All four were sampled at 100 year intervals for statistical analysis.

### 2.6. Statistical modelling

**Figure 2.**
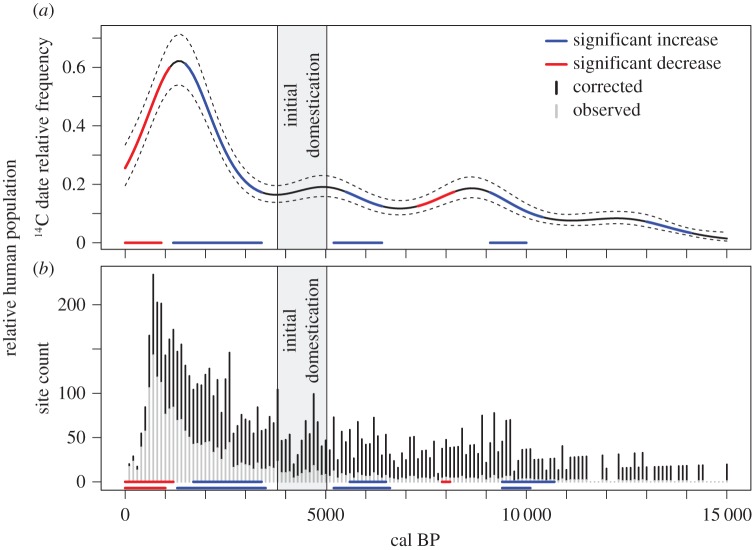
(*a*) Relative human population density as measured by a calibrated and taphonomically corrected KDE fit by a generalized additive model (GAM). The plot illustrates the model fit with 95% confidence intervals and is colour coded to indicate significant (*α* = 0.01) periods of increase (blue) and decrease (red) based on the first derivative of the model fit. The period of initial domestication is defined by the earliest occurrences of domestic *C. pepo* at 5025 cal BP, *H. annus* var. *macrocarpus* at 4840 cal BP, *I. annua* var. *macrocarpa* at 4400 cal BP, and *C. berlandieri* at 3800 cal BP. Periods of significant change in the taphonomically uncorrected GAM are shown as coloured horizontal lines at the base of the panel. (*b*) Histogram of relative human population levels through time as measured by observed and corrected archaeological site counts in 100 year intervals. Colour-coded horizontal lines at the base of the panel indicate periods of significant change in the taphonomically corrected (top) and uncorrected (bottom) GAM fits. As predicted by the model-based BE framework, human populations increase significantly to a local peak prior to initial domestication in the study area.

To examine whether the variation in these four proxies varies significantly through time, we fit each as a function of time using generalized linear (GLM) and generalized additive models (GAM; figure 2). Following Shennan *et al.* [26], we specified a Poisson distribution and log link relying on quasi-likelihood estimation. GAMs allow nonlinear fits between dependent (date density and site counts) and independent (time) variables using a penalized regression spline approach [27–29]. This allows the data to ‘speak for itself’ by increasing the degrees of freedom up to an optimal level while still maximizing parsimony. As a result, GAMs allow the independent variable(s) to explain a greater proportion of the dependent variable and to account for local variation in the relationship between the two variables. The optimal degrees of freedom (knots) are defined following generalized cross-validation [28,29]. Model results report the estimated degrees of freedom (edf), *r*^2^- and *p*-values. A GAM is useful in this instance because it allows for description of local fluctuations in population that may be missed by more strictly parsimonious models like a single polynomial GLM, which may better describe ‘global’ trends in population. Because the model will optimize the degrees of freedom, the fitted trend will also smooth over what are likely to be spurious fluctuations in the population data.

### 2.7. Identifying significant rates of change

In order to identify significant periods of change (increase or decrease) in the fitted trend, we relied on Simpson's [30–32] approach to estimate the first derivative of the GAM fit based on the finite difference between points (in this case, 100-year intervals) and then identify significant moments of change where the 95% confidence interval for the slope of the fit does not overlap with zero. When the first derivative, or to simplify, the rate of change of the GAM is significantly different from zero, human populations are either significantly increasing or decreasing. Following Contreras and Meadows [33], we then evaluate this approach using simulated population data in the electronic supplementary material, text S1.

All analyses were run in the R environment [34]. The complete dataset on corrected and observed model fits of radiocarbon dates and site counts is available as the electronic supplementary material, table S1.

## 3. Results

Sampling the CARD [18] using a standard deviational ellipse (*α* = 0.05) around the seven sites with the earliest evidence for domestication (figure 1) returns a total of 3750 dates that pass chronometric hygiene protocols. The distribution of median calibrated dates ranges from 50 to 15 423 cal BP and is heavily skewed towards the Late Holocene, with a first and third quartile of 747 and 2812 cal BP, respectively.

### 3.1. Diachronic trends in date density

Examining the distribution of dates with KDE (electronic supplementary material, figure S1) reveals a global trend of increasing populations through time (GLM: $R_{L}^{2}=0.81,$ *p* < 0.0001). This trend holds when the KDE is corrected for taphonomic loss (GLM: $R_{L}^{2}=0.65,$ *p* < 0.0001). Examining this variation using a generalized additive model (GAM) shows that local variation in both the observed (GAM: *r*^2^ = 0.76, edf = 8.053, *p* < 0.0001) and taphonomically corrected (GAM: *r*^2^ = 0.73, edf = 8.596, *p* < 0.0001) trends are explained by time (figure 2*a*).

Examining the first derivative of the taphonomically corrected GAM fit reveals long durations of stasis punctuated by six periods of significant population change (figure 2*a*). Four represent periods of significant population increase: 14 100–13 000, 10 400–9100, 6400–5500 and 3400–1500 cal BP; while two represent periods of significant population decrease: 8200–7300 and 1100–0 cal BP. Most importantly for the focus of this paper, and as predicted by the BE hypothesis, the period of initial domestication is preceded by a period of significant growth from 6400 to 5500 cal BP. From the trough in the model fit at approximately 6900 cal BP to the peak at 5200 cal BP, relative human populations roughly double in the region. An examination of the model residuals (electronic supplementary material, figure S2) shows that the GAM does well predicting population change throughout most of the record except for during the final two periods of significant change, where model predictions are less precise due to the high rate of change in population from 0 to 3000 cal BP, including the sharp peak at 700 cal BP (electronic supplementary material, figures S1 and S2) which is not adequately captured in the model fit.

Four of these six periods of significant change, as well as the broader trends, are similarly shown in the observed radiocarbon data when not taphonomically corrected (figure 2*a*; electronic supplementary material, figure S3). This demonstrates that the significant increase prior to initial domestication is not a result of the taphonomic correction process, but is an innate feature of the radiocarbon record. However, the significant increase in population from 14 100 to 13 000 cal BP and the significant decline in population from 8200 to 7300 cal BP shown in the taphonomically corrected model (figure 2) may be due to taphonomic correction, as they do not appear in the uncorrected model (figure 2*a*; electronic supplementary material, figure S3).

### 3.2. Diachronic trends in site counts

Examining the distribution of dated site counts shows that these trends are robust across alternative methods of evaluation (figure 2*b*). Site counts reveal a global trend of exponential increase (GLM: $R_{L}^{2}=0.80,$ *p* < 0.0001), even when corrected for taphonomic loss (GLM: $R_{L}^{2}=0.59,$ *p* < 0.0001). Observed (GAM: *r*^2^ = 0.78, edf = 7.77, *p* < 0.0001) and taphonomically corrected (GAM: *r*^2^ = 0.71, edf = 8.274, *p* < 0.0001) site counts also vary locally as a function of time (figure 2*b*).

Examining the first derivative of the GAM fit describing the taphonomically corrected number of sites reveals that populations increase significantly over three periods (10 700–9400, 6500–5600 and 3400–1700 cal BP) and decrease over two periods (8100–7900 and 1200–0 cal BP). The period of significant decrease between 8100 and 7900 cal BP does not appear in the model of observed site counts (figure 2*b*) and may therefore be an artefact of the taphonomic correction process. Importantly, the period of population increase prior to initial domestication remains significant across both corrected and uncorrected site counts (figure 2*b*; electronic supplementary material, figures S4 and S5).

The number of sites present in the CARD through time shows the same trends as the radiocarbon KDE model. The first derivative of the fit across all four proxies exhibit a significant increase in population preceding initial domestication, supporting the BE hypothesis of domestication.

## 4. Discussion

### 4.1. Increasing human populations prior to initial domestication

The observed trends suggest that populations in the study area were relatively high throughout the Middle Holocene and increased significantly to a local peak concurrent with the earliest date of initial domestication (5000 cal BP). Not only were populations at this time higher than ever before, but also they had been significantly increasing for approximately 1000 years, likely placing strong pressure on local resources. Between 5000 cal BP and the earliest dates for the full adoption of a crop complex at 3800 cal BP, populations then experienced a period of stasis, suggesting sustained pressure on the resource base through this time period. While the evidence does not indicate whether this population growth occurred *in situ* or was the result of immigration from outside the study area, both possibilities would result in the same outcome: population pressure leading to resource deficits that encourage domestication.

This pattern of high human populations that increased prior to 5000 cal BP aligns with the predictions of the model-based BE hypothesis of initial domestication. High population levels likely led to local declines in foraging return rates, to which individuals responded optimally by expanding diet breadth in order to take lower profitability resources and eventually manipulate these resources. With broader diets that included more abundant but lower profitability resources, populations should grow at the same rate, but up to a higher carrying capacity provided by the broader resource base that allows for more individuals within a given area [35–38]. Given broader diets, lower foraging returns, and more people, we expect individuals would have placed selective pressure on certain plant resources. While this may have been unintentional at first, individuals may have eventually begun to intentionally alter plants in ways that led to increased yields and reduced processing costs, ultimately resulting in domestication. The prediction of the NC hypothesis, that there should be low populations and no population increases before the time period of initial domestication, is not supported by these data.

These findings are consistent not only with predictions we derive from foraging theory, but also with other archaeological data. Anderson [39] evaluated changes in the number and location of archaeological sites through time in the Southeastern USA and identified an increase in site numbers from the Middle to the Late Holocene. In the Duck River Valley of Tennessee, near the Hayes site, Miller [40] makes use of the number of archaeological sites within physiographic sections of the river valley to show that populations increased from the terminal Pleistocene through the Late Holocene. In line with predictions from an ideal free distribution model [41], he argues that infilling of people occurred in this region through time, suggesting that populations were increasing prior to initial domestication. Lending support to our findings, Miller interprets his results as indicating that a warming climate in the Middle Holocene increased the abundance of shellfish, oak, hickory and deer. Miller argues that foragers narrowed their diets to focus on these resources, but once these positive climatic effects ended in the Late Holocene and the abundance of these resources declined, individuals living at high population densities at this time were driven to intensively exploit the low-return plants that would become the Eastern Agricultural Complex.

Based on the observed high and increasing populations, we predict low foraging efficiency prior to initial domestication, potentially as a result of increased competition or overexploitation of high-return resources, but future work is needed to evaluate this. Styles & Klippel [42] document a broad trend of declining white-tailed deer and increasing fish exploitation through time in the region which may be suggestive of declining foraging efficiency and increasing intensification; however, the exact relationship between such changes and initial domestication remains unanalysed. It must be emphasized that our results do not demonstrate low foraging efficiency anywhere in the study area, only the increased human population levels that may precede and cause such a phenomenon out of increased resource exploitation and competition. An analysis of faunal and botanical data from sites in the study area, specifically those seven from which the earliest domesticates have been recovered, may provide the data needed to address this question.

In addition to an absolute increase in human impact on the environment, it is also possible that the increased population levels documented here brought about decreased mobility and smaller foraging territories, thereby amplifying the *per capita* impact on local resources leading to initial domestication in the region [43], though this remains to be assessed as well. Given this possibility, settlement pattern data may elucidate trends in population packing that could have led to population–resource imbalances.

Research is also needed to determine the environmental setting in which this predicted process of resource depression and domestication occurs. The sites of earliest domestication are predominately located in lowland river valleys, with the exception of the Marble Bluff, Cloudsplitter and Newt Kash rockshelters. This observation has been used to argue that domestication first occurred in floodplain settings in North America [19]. Others, however, claim that an upland origin is just as likely [43,44]. Ongoing work illustrating where these observed periods of population growth are concentrated, coupled with detailed analyses of subsistence data, will help determine whether the initial domestication events occurred through experimentation in floodplain settings [19,45], or out of necessity in upland habitats [43,46].

### 4.2. Population–resource imbalance as a global driver of domestication

While our analysis reveals significant population increases in Eastern North America that may have led to declines in *per capita* foraging return rates through increased competition [41] or resource depression [47], it is important to note that population growth is a sufficient, but not a necessary condition for disequilibrium between populations and resources that may precipitate initial domestication. It is entirely possible that, holding population constant, local ecological changes that reduce the abundance of profitable resources could lead to the same population–resource imbalance, as evidenced in other domestication events around the world.

Illustrating the effects of population increases on foraging efficiency, domestication in Southwest Asia follows a long period of ‘broad spectrum’ foraging [48] resulting from population increases and subsequent reductions in encounters with high-ranked prey [5,49–53]. On the opposite side of this imbalance, domestication events in the Neotropical lowlands of Central and South America [54] and Northern China [55,56] do not reveal evidence for high or increasing human populations prior to domestication; yet, these events are still due to deficits in the food supply relative to demand. In the Neotropical case, environmental changes at the Pleistocene/Holocene transition are argued to have lowered encounter rates with high-ranked prey items and thus lowered foraging return rates [57,58]. In Northern China, hunter–gatherer populations prior to domestication were low and highly mobile, but still experienced resource shortages that encouraged intensified foraging leading to the manipulation of wild millet [55,59]. Such environmental changes in resource availability result in population–resource imbalances just as an increase in population would, though by affecting the resource base instead of population levels.

### 4.3. Integrating behavioural ecological models and niche construction

For simplicity, we refer to the two competing explanations of initial domestication in this paper as the NC and BE hypotheses, but for the sake of clarity it is important to note that the specific predictions tested here are two of many that could potentially be drawn from these larger bodies of theory to explain domestication. Support or falsification of one of these hypotheses therefore does not necessarily constitute support or refutation of NC and BE as overarching bodies of theory, but of the specific predictions derived from these frameworks.

It is furthermore important to recognize the inconsistencies between the use of NC in biology and in anthropology. While the biological literature is also rife with disagreements between practitioners of BE and advocates of NC theory [60], these debates are more focused on whether NC is a unique evolutionary process or simply part of standard evolutionary theory [61,62]. Unlike in anthropology [2,3,5,45], researchers on both sides of the debate in biology agree that NC ‘does not make an unambiguous and clear prediction about the natural world’ [60]. This stands in contrast to studies of initial domestication, in which researchers oppose simple models that help generate predictions about why individuals would benefit from niche constructing behaviour. In order to explain why an organism would modify their environment, researchers therefore need to draw on evolutionary theory that can make predictions about how individuals are expected to behave in particular environmental circumstances [10].

As such, we contend that the BE-derived hypothesis of initial domestication is not only better supported by the available archaeological data, but is also better equipped to generate theoretically grounded and testable predictions than is the NC hypothesis. This does not mean, however, that there is no need to pay attention to NC. Explaining variation in human behaviour also certainly requires understanding the dynamic processes of habitat modification and ecosystem engineering [63] that are the focus of NC theory. For example, evidence of anthropogenic NC in the Late Holocene of Eastern North America [64,65] requires explaining why people engaged in such niche constructing behaviour and what the effects of that behaviour were on subsequent generations. These patterns of habitat modification probably occur for reasons best predicted by foraging theory models derived from BE, which provide a framework in which researchers can generate testable predictions based on logic from economic and evolutionary theory [10]. But the long-term impacts of this anthropogenic habitat modification also require understanding that subsequent generations inherited an altered environment that would change subsistence decisions. We conclude, as have others [66], that NC can and should be integrated with foraging theory under the umbrella of BE in order to best understand the evolutionary motivations behind human behaviours.

### 4.4. Potential sources of error

Given the potential risk of drawing spurious conclusions from this dataset, an exploration of possible confounding factors is necessary. Here, we briefly discuss bias that may be introduced from the CARD, differential preservation, modelling and researcher bias [23].

As stated previously, we obtained only unique radiocarbon dates from the CARD, eliminated any date from geological and palaeobiological contexts, and did not include any dates marked as ‘anomalous' in the database. Our dataset is therefore restricted to those dates associated with human activity and consistent with other contextual information (stratigraphic, typological, etc.) as determined by the original researcher who supplied a given date to the CARD. While these decisions reduce the risk of inaccurate analyses, other problems remain. Radiocarbon dates from several decades ago are listed in the CARD as are dates with large standard errors. The error inherent in these kinds of dates could introduce inaccuracies in our model. However, the periods of significant change we observe in human populations through time are almost all several centuries or millennia in duration. The decadal-scale error that may result from imprecise dates would be unlikely to substantially alter our model to the point where thousand-year-long periods of significant change were impacted.

Differential preservation of organic materials may also impact the frequencies of radiocarbon dates through time. Certain contexts such as shell middens, rockshelters and caves create conditions more amenable to the preservation of organic remains. More abundant radiocarbon dates for a given time period may therefore result not from increased human populations, but from increased dating of abundant sites with a high degree of organic preservation. Of these three site types, shell middens are particularly abundant around the time of initial domestication in Eastern North America, particularly in the Southeast. At least in certain parts of the region though, shellfish exploitation actually peaked several millennia after initial domestication occurred [67]. If our radiocarbon or site-based models of human population were heavily impacted by the abundance of shell middens preserving more organic remains, the GAM should not peak locally at 5000 cal BP as it does, but probably between 2000 and 4000 cal BP. The greater degree of organic preservation afforded by shell middens is therefore not likely to substantially affect our results.

Error in interpretation may also result from the way population proxies are analysed. Here, we undertook a novel approach using GAMs to fit diachronic population trends, and the first derivatives of these fits to evaluate significant periods of change. This method is useful in that it balances parsimony and goodness of fit, but there is a possibility that these models could identify spurious periods of change that result not from differences in human populations, but from differences in the sample of radiocarbon dates [33]. While simulations show that this method can produce spurious results using site density, false positives are extremely rare using histograms of counts (see the electronic supplementary material, text S1). Given that our empirical results are consistent across both the KDE method examining date density and the histogram method examining site counts, we find the conclusions to be real and robust.

An additional problem with the use of radiocarbon dates as a proxy for human population levels may be found in the form of researcher bias [23]. Certain time periods, geographical regions or site types may be preferentially investigated by archaeologists, leading to differential dating through time and space that reflects the actions of scientists, not past human population levels. This problem continues to plague radiocarbon-based population measures. While some solutions have been proposed to control for researcher bias in geographical sampling intensity [68], a way to control for temporal biases in sampling has yet to be proposed. We therefore acknowledge that it is possible that our results may be impacted by differential investigation of various time periods, geographical regions, site types and archaeological contexts. With 3750 radiocarbon dates from a large region and lengthy time span, we expect such biases to be minimal, but cannot rule them out.

Finally, these methods may also be capturing false periods of change that result from peaks or plateaus in the calibration curve [69]. If periods of inferred population change covary with periods of time in the calibration curve, models may show inaccurate fluctuations or periods of stasis that result from the calibration curve, not from past human population change or stability. However, Brown [69] has shown that such systematic error does not typically lead to false peaks in sampled radiocarbon records, and no problematic peaks and troughs exist in the calibration curve during the millennia surrounding initial domestication at 5000 cal BP [22].

## 5. Conclusion

The results of this analysis align with the predictions derived from the BE hypothesis for initial domestication in Eastern North America: populations were high and increased significantly prior to the beginnings of domestication at 5000 cal BP, doubling over the course of the preceding 1700 years. This suggests that imbalance between populations and resources, manifested as low foraging efficiency, drove the shift towards domestication. It is possible that wild foods were not able to sustain high human populations in the Middle and Late Holocene, making domestication an optimal response to low foraging profitability. It is also possible that increasing populations led to decreased territory size [43], excluding foragers from profitable patches and leading to low foraging efficiency.

Within this framework, declining foraging returns are likely to be the most immediate motivation to initiate the intensification processes that precedes domestication. Regardless of whether populations increase or resource availability decreases, such deficits should either unintentionally alter the selective pressures humans place on wild plants or encourage the intentional manipulation of plants in order to reduce processing time and increase yields. In Eastern North America, it is the case that populations increased prior to domestication, but we do not expect this to be a universal trend. What we do predict to be universal, however, is the disequilibrium between supply and demand. Humans are unlikely to put significant enough pressure on wild resources unless there is a need to do so. Profitable wild foods must be scarce enough relative to human population levels before it would be worthwhile for individuals to pay the costs of investing time and energy into the process of domestication without guaranteed future rewards. Further investigation of these patterns in line with predictions derived from foraging theory and niche construction theory is needed, and this work should integrate both perspectives within BE. We expect that such investigations will find a general pattern of population–resource imbalance preceding domestication that can help explain the timing and process of domestication wherever it happened in the world.

## Supplementary Material

Text S1: A series of simulations using idealized population data that was randomly sampled to represent radiocarbon dates in order to validate the methodological approach used in this paper.

## Supplementary Material

Dataset S1. Data of corrected and observed summed probability distributions (SPDs), site counts, and generalized additive model (GAM) fits per 100 year intervals from 0–15,000 cal BP.

## Supplementary Material

Figure S1. Kernel density summed probability distribution (SPD) plots with 95% confidence intervals following the Sheather-Jones method (60) of calibrated median radiocarbon dates for raw and taphonomically corrected (52) dates.

## Supplementary Material

Figure S2. Unadjusted residuals for each 100 year interval showing the difference between values of the summed probability distributions (SPDs) and the fitted values of the generalized additive models (GAMs) for observed and taphonomically corrected radiocarbon dates. GAM fits are less accurate from 0–1,000 cal BP given the high rate of change in the SPDs.

## Supplementary Material

Figure S3. Relative population density from a calibrated but not taphonomically corrected (52) summed probability distribution of radiocarbon dates through time fit with a generalized additive model (GAM). The plot illustrates the model fit with confidence intervals and is color coded to indicate significant (a=0.01) periods of increase (blue) and decrease (red).

## Supplementary Material

Figure S4. Relative population density from a calibrated and taphonomically corrected (52) summed probability distribution (SPD) of site counts through time fit with a generalized additive model (GAM). The plot illustrates the model fit with confidence intervals and is color coded to indicate significant (a=0.01) periods of increase (blue) and decrease (red).

## Supplementary Material

Figure S5. Relative population density from a calibrated but not taphonomically corrected (52) summed probability distribution (SPD) of site counts through time fit with a generalized additive model (GAM). The plot illustrates the model fit with confidence intervals and is color coded to indicate significant (a=0.01) periods of increase (blue) and decrease (red).

## Data accessibility

All data from the models presented in this paper are available as the electronic supplementary material (dataset S1). Radiocarbon dates and site counts were obtained from the CARD on 25 November 2015 and are available to researchers with permission from the administrators of the CARD.
