# Supplementary material for: Population growth as a driver of initial domestication in Eastern North America
Source: R Soc Open Sci. 2016 Aug 3;3(8):160319. doi: 10.1098/rsos.160319 (PMC5108960; doi:10.1098/rsos.160319)
Supplement: Text S1: A series of simulations using idealized population data that was randomly sampled to represent radiocarbon dates in order to validate the methodological approach used in this paper. [file rsos160319supp1.pdf]

Citation: Weitzel, E. M. and B. F. Codding. Population growth as a driver of initial domestication in Eastern North America. *Royal Society Open Science*.

Supplementary material

Text S1: Simulations

In order to validate the methodological approach used in this paper, we ran a series of simulations using idealized population data that was then randomly sampled to represent radiocarbon dates. This process is described below in five steps, followed by an analysis of the simulated results. As in the paper, all analyses were completed in the R environment (1).

Step 1: Generate idealized population data

First, population data were generated based on a logistic differential equation:

$$\frac{dN}{dt} = rN\left(1 - \frac{N}{K}\right)$$

The population at any one time (N_t) can be estimated as:

$$N_t = \frac{N_0 K e^{-rt}}{K + N_0(e^{rt} - 1)}$$

where K is the carrying capacity, N_0 is the initial population, r is the population growth rate and t is the time interval of interest. Two basic hypothetical population curves were generated; one characterizing long-term population stasis, and another characterizing long-term population growth (Text S1 Fig. 1). The former was specified with an initial population of 0.5 and an annual growth rate of zero. The latter was specified with an initial population (N_0) of 0.001 and an annual growth rate (r) of 0.001. In both cases, the carrying capacity was set to one. Each permutation was evaluated over a window of time (t) from 0 to 15,000 years, consistent with the data analyzed in this paper. We also provide a third and slightly more complex approximation of populations over the 15,000 year window by specifying zero growth for the first 5000 years followed by logistic growth for the remaining 10,000 years. This hypothetical population sequence is also evaluated as a third test of this approach. As a first examination, the ideal populations were fit using the generalized additive model (GAM) approach described in the paper. As a more accurate examination, these idealized population data were then sampled to generate distributions representative of radiocarbon dates.

Step 2: Generate dates from idealized population data

Following Contreras and Meadows (2), we randomly sampled these ideal populations with replacement to generate a representative distribution of dates. This approach translates observed or idealized population data into a distribution of expected dates that would be created if the archaeological record of radiocarbon dates were a representative sample of human activity (which is the basis of the "dates as data" approach; see Rick [3]). In keeping with the sampling intensity represented in our data of 0.25 dates per year, we randomly sampled 3750 dates across the 15,000 year period. These data were then described following the two approaches used in this paper, specifically by a kernel density function and by a histogram.

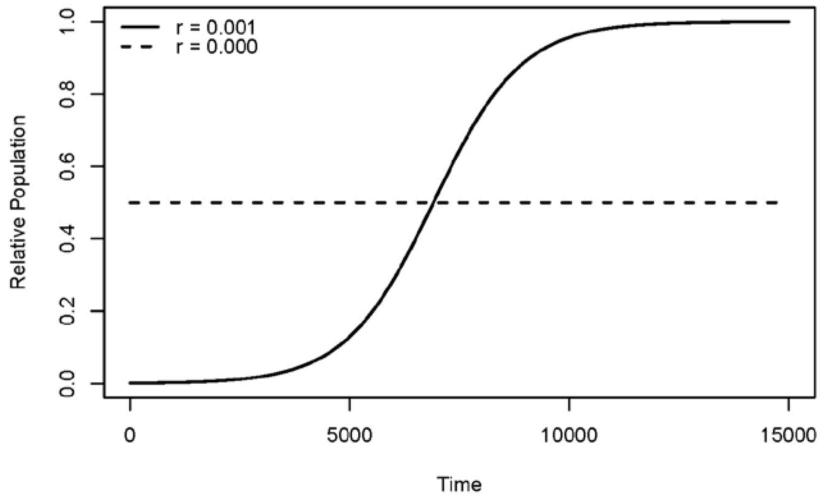

Text S1, Figure 1. Idealized population data illustrating long-term population growth (solid line) compared with zero population growth (dashed line). Note that the population increases with time because the x-axis represents time (t) in the logistic growth equation, not years before present.

Step 3: Describe the dates as a density function and histogram

The distribution of the random sample of dates derived from each of the idealized populations was described using kernel density estimation with a specified bandwidth following the Sheather-Jones (4) method sampled in 100-year intervals, and using a histogram with 151 breaks providing estimates at 100-year intervals over the 15,000 year sequence. These are the same approaches used to analyze the empirical data in this paper, except that the empirical histogram approach removed repeated radiocarbon dates from the same site in each 100-year interval (see Methods).

Step 4: Fit data with a generalized additive model and evaluate derivatives

As in the paper, these distributions were fit using a generalized additive model from the mgcv package in R with the number of knots estimated following the generalized cross validation criterion (5, see Methods). The model fit was then evaluated using Gavin Simpson's (6) derivative approach to identify periods of significant population increase or decrease (see Methods).

Step 5: Iterate

In order to generate a large enough sample of evaluations, steps 1-4 were implemented in R as a user-defined function that was nested in the replicate function to generate 1000 iterations of each permutation. The results of these simulations were plotted and the output data summarized to evaluate each approach. For visualization purposes, simulated plots show the y-axis as the relative population normalized by the median interquartile range of the dates sampled in each iteration.

Results

Idealized data

Generalized additive models fit the idealized population data “perfectly”: the first derivatives of the model fits accurately show significant increases in population through time with the growing population and accurately show no moments of population increase or decrease with the zero growth population. If these trends are not biased by sampling and summary with either kernel density functions or histograms, then these results should remain consistent.

Sampled Data: Zero Growth Population

As with the idealized data, the zero growth population should produce no periods of significant change.

Kernel Density. The density function approach indicated false significant decreases and increases for all 1000 runs. The average 100-year window had a 30.99% chance of falsely being declared as significantly declining and a 30.99% chance of being falsely declared as significantly increasing. Shown in Text S1 Figure 2a, these simulations suggest that the kernel density approach is prone to error when examining long-term zero growth. The high number of false positives may result from the limited variation in the sampled uniform distribution which the kernel density estimation “over-fits”.

Histogram. The histogram approach fared much better. Only 16 out of 1000 runs (1.6%) identified false increases with a less than 0.1% chance that any 100-year window would be falsely identified as increasing. Fifteen out of 1000 runs (1.5%) identified false decreases, with a less than 0.1% chance that any one 100-year interval would be falsely flagged as significantly decreasing. These results show that the histogram approach is robust to errors caused by sampling and description. These results are shown in Text S1 Figure 2b.

Sampled Data: Growing Population

The growing population should produce long periods of significant population increase with no periods of population decline.

Kernel Density. Sampling a growing population, all 1000 runs using the kernel density method correctly identified a long period of population growth, but all 1000 also identified false declines. Any 100-year interval had a 72% chance of being correctly identified as increasing, and a 15% chance of being falsely identified as declining. Most of the latter result from artificial declines in the last few thousand years of the sequence (Figure 2c), due to the density function poorly fitting the data at the extremes of the sequence.

Histogram. Using the histogram method, all 1000 iterations correctly identified the substantial period of growth throughout the sequence (Figure 2d). Only 16 of 1000 iterations incorrectly identified a period of declining populations, with any one 100-year interval having a less than 0.1% chance of resulting in a false positive.

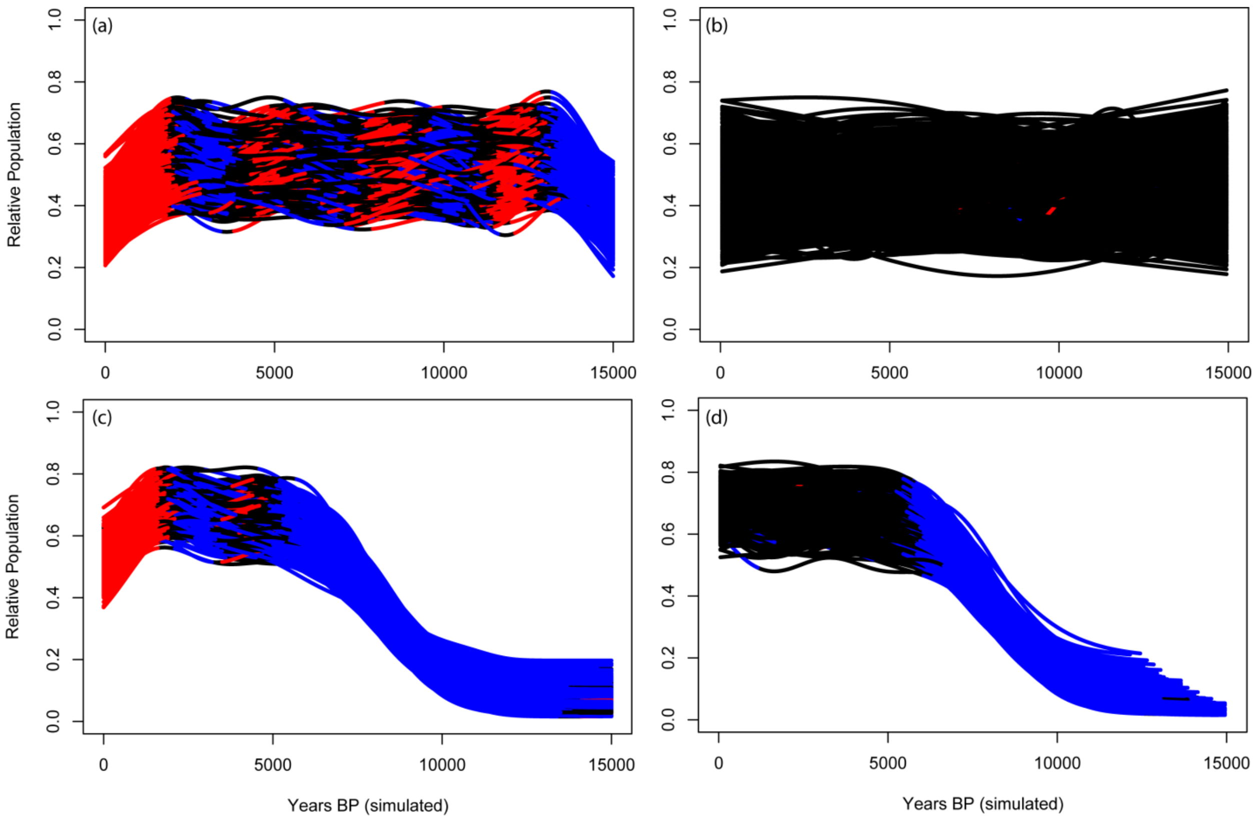

Text S1, Figure 2. Simulated evaluations of generalized additive model fits of randomly sampled data from idealized zero growth populations (top row) and growing populations (bottom row) using the kernel density approach (left column) and the histogram approach (right column) over 1000 iterations each. As in the empirical analysis, periods of significant population increase are coded in blue, periods of significant decrease are coded in red and periods not identified as significant change are in black.

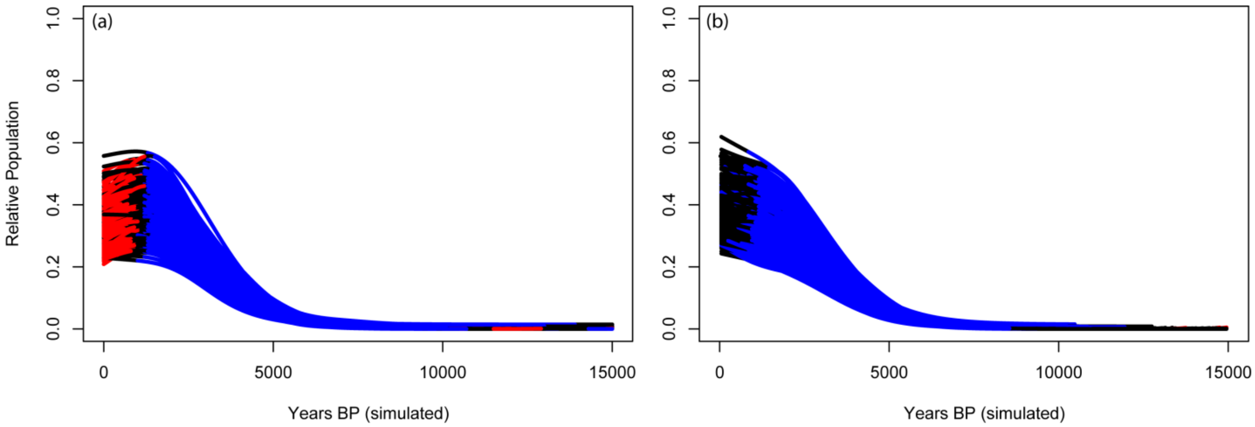

Text S1, Figure 3. Simulated evaluations of generalized additive model fits of randomly sampled data from a hypothetical population sequence that represents zero growth for the first 5,000 years (15,000-10,000 BP), followed by logistic growth for the remaining 10,000 years using the kernel density (left) and histogram (right) approaches. Lines are color coded as in Text S1 Figure 2.

Sampled Data: Hypothetical Population Sequence

If these approaches accurately represent the hypothetical population sequence, there should be no significant changes prior to 10,000 years ago followed by a significant population increase.

Kernel Density. Over 1000 iterations, the kernel density approach produced 649 runs wherein populations significantly increased prior to 10,000 BP, though the majority of the 100-year intervals responsible for these false positives were just prior to the actual period of initial increase (10,000 BP). Two hundred and eighty nine runs falsely identified periods of decrease prior to 10,000. But in both cases, the chances any one 100-year period prior to 10,000 would be falsely flagged as increasing or decreasing were low, 11% and 6% respectively.

Histogram. The histogram approach identified false increases prior to 10,000 BP in 701 out of the 1000 iterations, with any single 100-year period having a 11% chance of being identified as significantly increase, though the vast majority of these intervals were immediately prior to the post 10,000 BP period of actual growth. Only 17 out of 1000 runs identified false decreases during the pre-10,000 BP period of zero population growth, with an individual 100-year interval having only a 0.3% chance of being falsely flagged as significantly declining.

Discussion

The kernel density approach is much more sensitive to sampling error, making it prone to spurious results. These are likely caused by "artifacts" generated by the density function. Because density estimates trend toward zero at the beginning and end of the sequence, there are many more false positives in these regions. The high number of false positives returned by the kernel density estimation of the zero growth sequence likely result from the limited amount of variation across the entire data set (0.5 across 15,000 years), to which the density function responds by trying to over-fit the limited variation produced by sampling error. This conclusion is supported by the analysis of the hypothetical population sequence, which has much more variation over the 15,000-year sequence and to which the kernel density estimation responds with many fewer false positives. This suggests that the KDE approach, while generally accurate for describing significant periods of change in sequences with substantial variation away from the minimum and maximum, should be considered suspect unless if other methods provide corroborating evidence.

The histogram method is much more robust, rarely identifying false positives even at a sampling intensity of 0.25 dates per year. Compared with the KDE approach, this difference probably results because the binning or histogram approach does not carry lagged momentum like the kernel density approach; therefore it produces very few "artifacts" that will lead to spurious results. Importantly, the histogram approach is very unlikely to identify false periods of significant change when there is no actual change in a population, and is very likely to capture periods of significant population increase.

The results of this exercise suggest that researchers should use multiple approaches to characterize periods of change in the radiocarbon record. When results are consistent across multiple methods, they should be considered robust. While we examine sampling error here, additional modeling work could further clarify the benefits and problems of different approaches, including error that may result from variation in the calibration curve (7).

Citation: Weitzel, E. M. and B. F. Codding. Population growth as a driver of initial domestication in Eastern North America. *Royal Society Open Science*.

References

1. R Core Team. A language and environment for statistical computing. Vienna, Austria: R Foundation for Statistical Computing, 2015.

2. Contreras DA, Meadows J. Summed radiocarbon calibrations as a population proxy: a critical evaluation using a realistic simulation approach. Journal of Archaeological Science. 2014; 52:591-608.

3. Rick JW. Dates as data: an examination of the Peruvian preceramic radiocarbon record. American Antiquity. 1987; 1:55-73.

4. Sheather SJ, Jones MC. A reliable data-based bandwidth selection method for kernel density estimation. Journal of the Royal Statistical Society. Series B (Methodological). 1991; 53:683-90.

5. Wood SN. Fast stable restricted maximum likelihood and marginal likelihood estimation of semiparametric generalized linear models. Journal of the Royal Statistical Society: Series B (Statistical Methodology). 2011; 73(1):3-6.

6. Simpson G. Derriv.R [Internet]. 2015. Available from: https://gist.github.com/gavinsimpson/e73f011fdaaab4bb5a30

7. Brown WA. Through a filter, darkly: Population size estimation, systematic error, and random error in radiocarbon-supported demographic temporal frequency analysis. Journal of Archaeological Science. 2015; 53:133-47.
