# Supplementary figures and images for: Population growth as a driver of initial domestication in Eastern North America
Source: R Soc Open Sci. 2016 Aug 3;3(8):160319. doi: 10.1098/rsos.160319 (PMC5108960; doi:10.1098/rsos.160319)

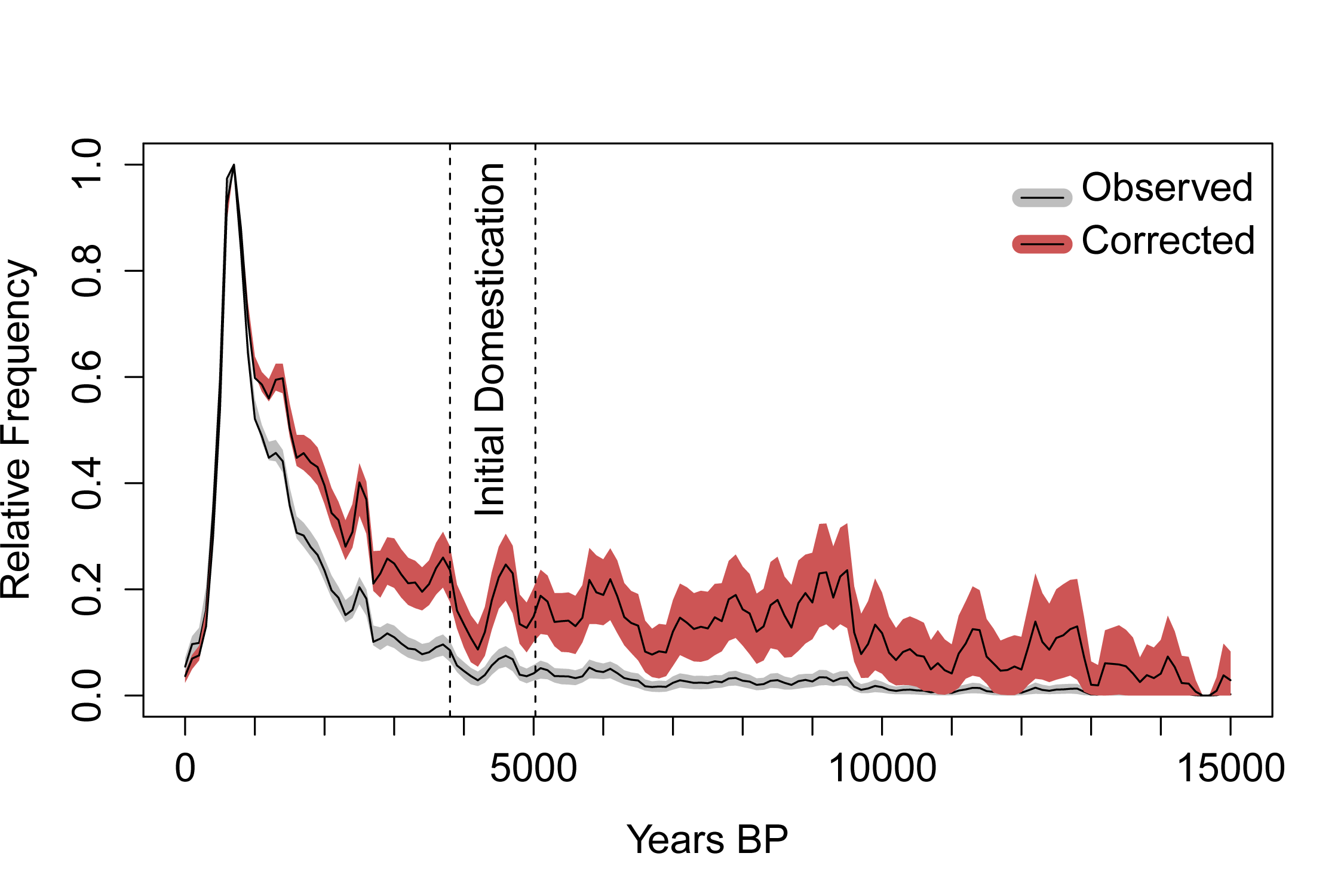

Supplement: Figure S1. Kernel density summed probability distribution (SPD) plots with 95% confidence intervals following the Sheather-Jones method (60) of calibrated median radiocarbon dates for raw and taphonomically corrected (52) dates. [file rsos160319supp3.png]

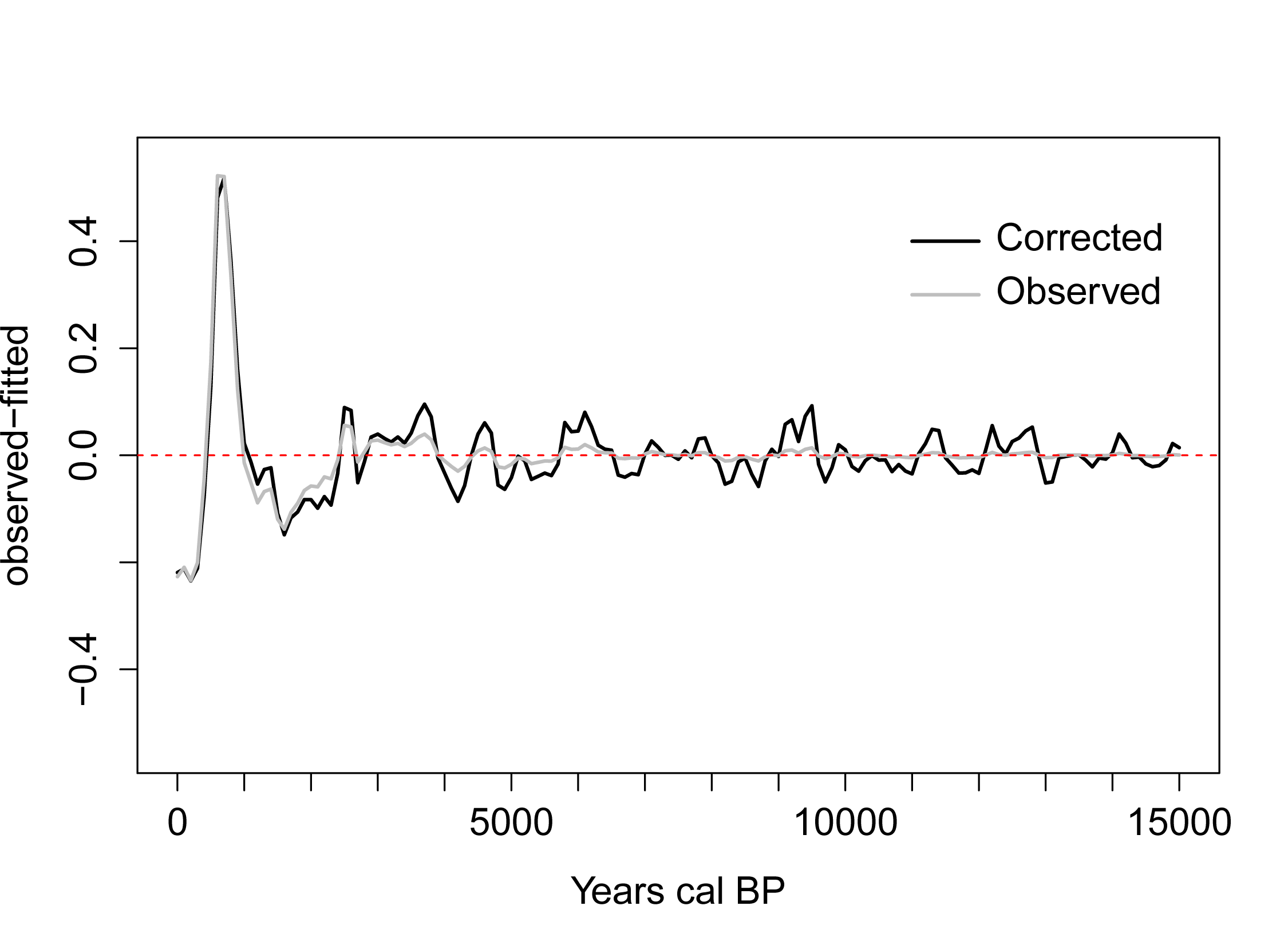

Supplement: Figure S2. Unadjusted residuals for each 100 year interval showing the difference between values of the summed probability distributions (SPDs) and the fitted values of the generalized additive models (GAMs) for observed and taphonomically corrected radiocarbon dates. GAM fits are less accurate from [file rsos160319supp4.png]

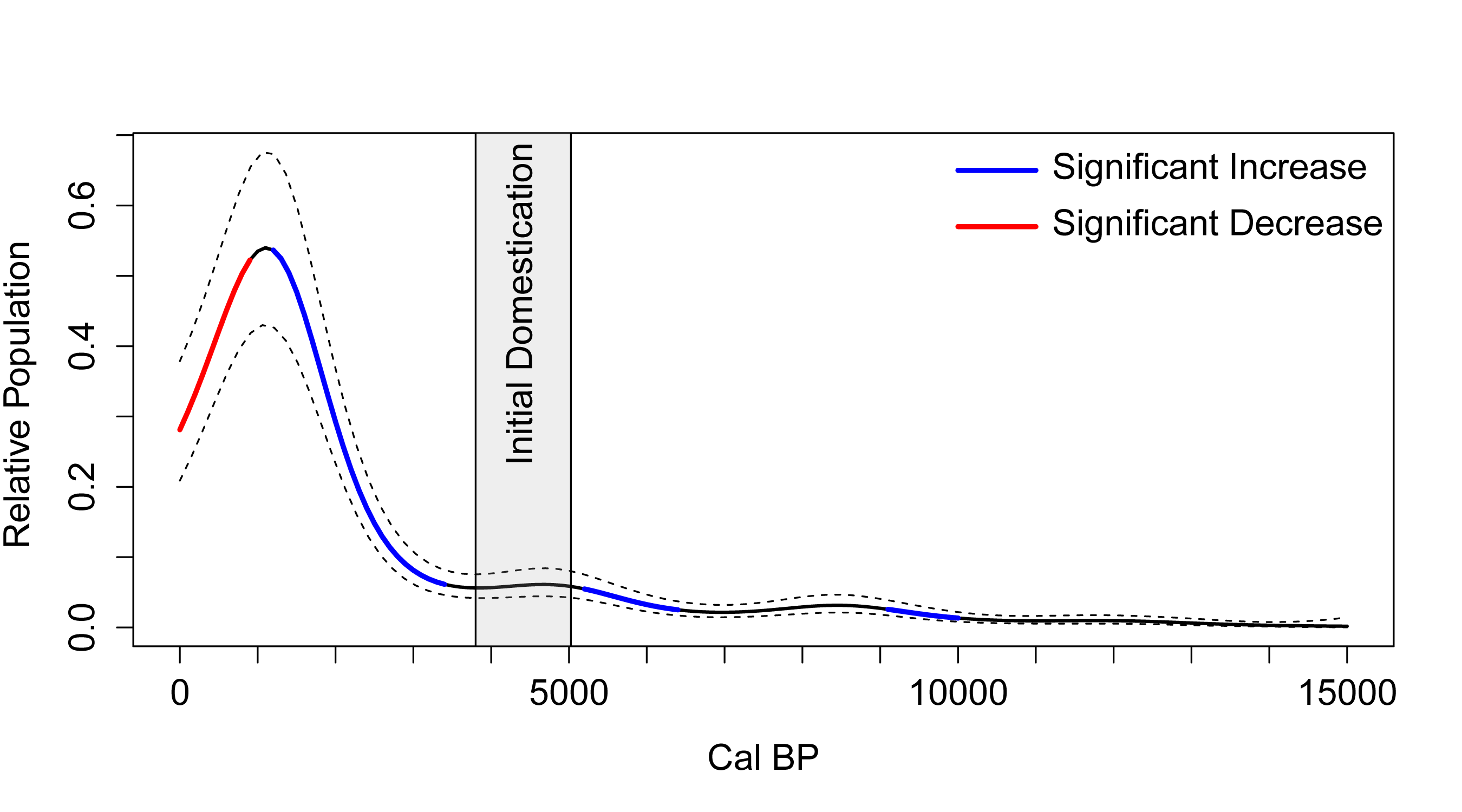

Supplement: Figure S3. Relative population density from a calibrated but not taphonomically corrected (52) summed probability distribution of radiocarbon dates through time fit with a generalized additive model (GAM). The plot illustrates the model fit with confidence intervals and is color coded to indicate si [file rsos160319supp5.png]

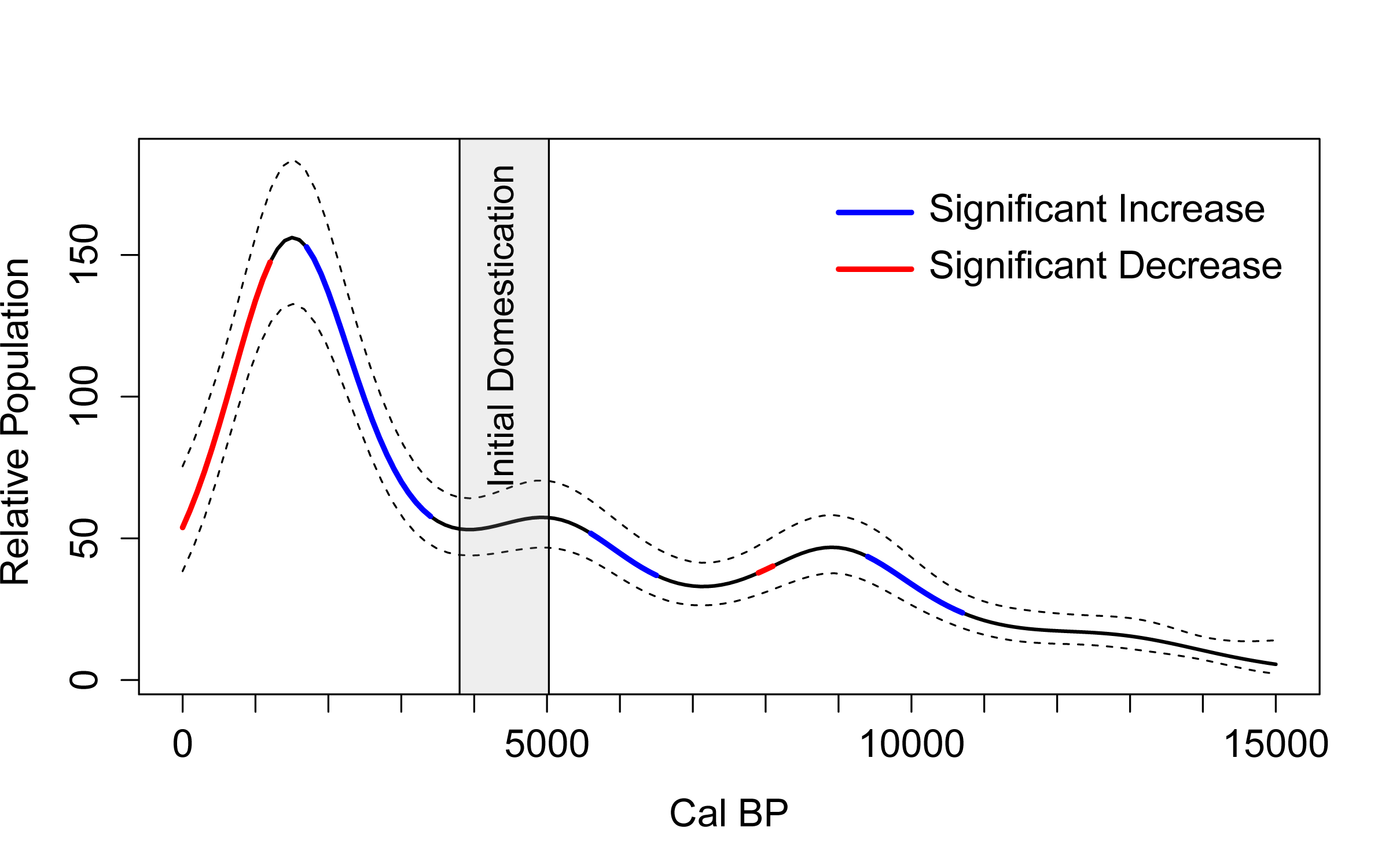

Supplement: Figure S4. Relative population density from a calibrated and taphonomically corrected (52) summed probability distribution (SPD) of site counts through time fit with a generalized additive model (GAM). The plot illustrates the model fit with confidence intervals and is color coded to indicate signif [file rsos160319supp6.png]

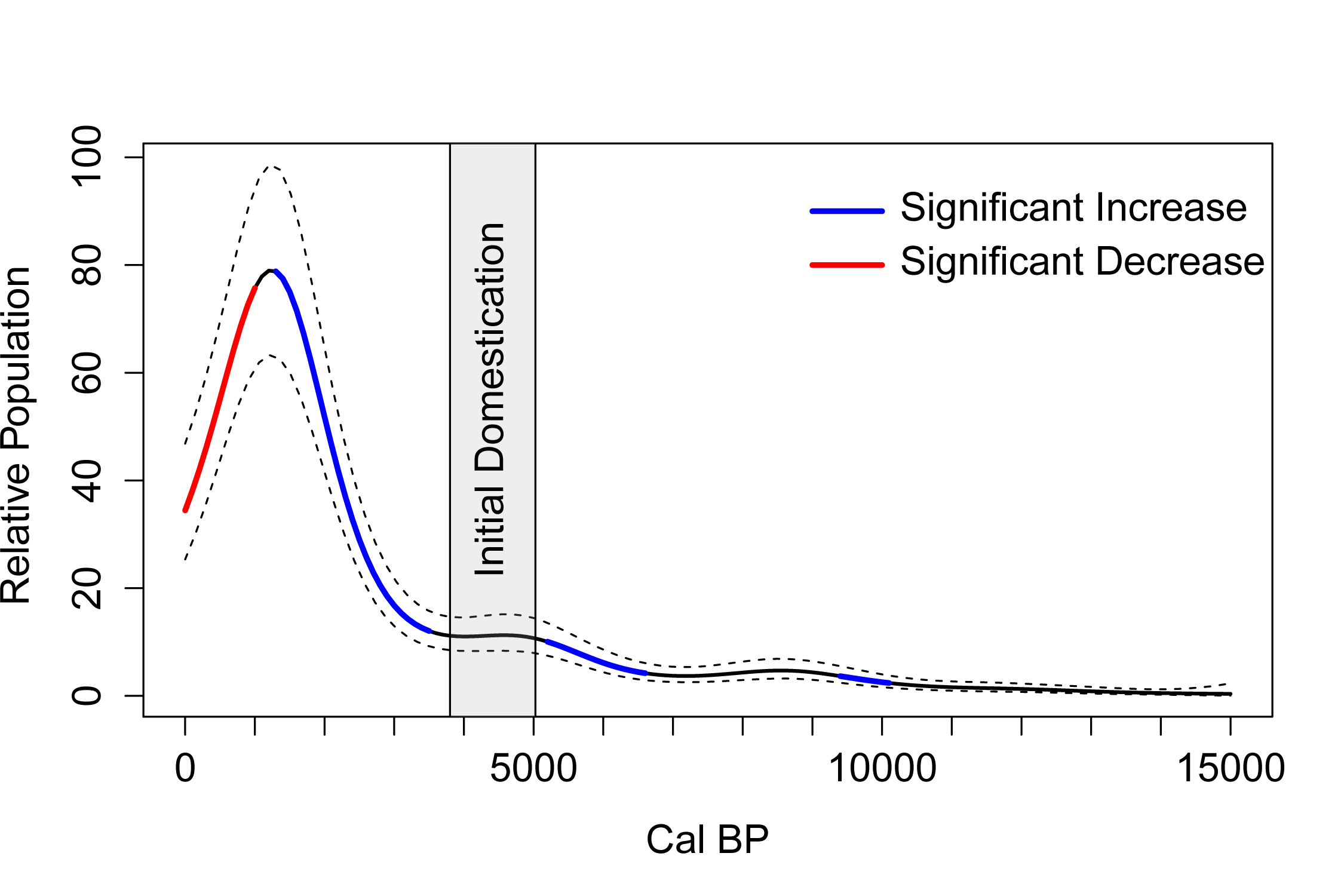

Supplement: Figure S5. Relative population density from a calibrated but not taphonomically corrected (52) summed probability distribution (SPD) of site counts through time fit with a generalized additive model (GAM). The plot illustrates the model fit with confidence intervals and is color coded to indicate si [file rsos160319supp7.png]
